# Phytochemical characterization of turnip greens (*Brassica rapa ssp*. *rapa*): A systematic review

**DOI:** 10.1371/journal.pone.0247032

**Published:** 2021-02-17

**Authors:** Gordana M. Dejanovic, Eralda Asllanaj, Magda Gamba, Peter Francis Raguindin, Oche Adam Itodo, Beatrice Minder, Weston Bussler, Brandon Metzger, Taulant Muka, Marija Glisic, Hua Kern

**Affiliations:** 1 Faculty of Medicine, Department of Ophthalmology, University of Novi Sad, Novi Sad, Serbia; 2 Department of Epidemiology, Erasmus MC, University Medical Center Rotterdam, Rotterdam, the Netherlands; 3 Institute of Social and Preventive Medicine (ISPM), University of Bern, Bern, Switzerland; 4 Swiss Paraplegic Research, Nottwil, Switzerland; 5 Public Health & Primary Care Library, University Library of Bern, University of Bern, Bern, Switzerland; 6 Nutrition Innovation Center, Standard Process Inc., Kannapolis, NC, United States of America; Higher Institute of Applied Sciences and Technology of Gabes University of Gabes, TUNISIA

## Abstract

**Objective:**

The *Turnip* (*Brassica rapa L*. *ssp*. *rapa*) is a leaf and root vegetable grown and consumed worldwide. The consumption of Turnip has been associated with beneficial effects on human health due to their phytochemicals that may control a variety of physiological functions, including antioxidant activity, enzyme regulation, and apoptotic control and the cell cycle. The current systematic review of the literature aims to evaluate both the profile and quantity of phytochemicals commonly found in Turnip greens and to provide perspectives for further investigation.

**Methods:**

This review was conducted following the PRISMA guidelines. Four bibliographic databases (PubMed, Embase, Web-of-Science and Cochrane Central Register of Controlled Trials) were searched to identify published studies until April 8th, 2020 (date last searched) without data and language restriction. Studies were included if they used samples of Turnip greens (the leaves), and evaluated its phytochemical content. Two reviewers independently evaluated the titles and abstracts according to the selection criteria. For each potentially eligible study, two reviewers assessed the full-texts and independently extracted the data using a predesigned data extraction form.

**Results:**

Based on the search strategy 5,077 potentially relevant citations were identified and full texts of 37 studies were evaluated, among which 18 studies were eligible to be included in the current review. The majority of included studies were focused on identification of glucosinolates and isothiocyanates (n = 14, 82%), four studies focused on organic acids, and five studies reported phenolic component profile in Turnip greens. Among included studies nine studies (50%) provided information on phytochemical’s content. We found 129 phytochemicals (19 glucosinolates, 33 glucosinolate-breakdown products, 10 organic acids and 59 polyphenolic compounds) reported in Turnip greens. Flavonoids were mainly present as quercetin, kaempferol and isorhamnetin derivatives; while aliphatic forms were the predominant glucosinolate (gluconapin was the most common across five studies, followed by glucobrassicanapin). In general, the phytochemical content varied among the leaves, tops and Turnip roots.

**Conclusions:**

Emerging evidence suggests the Turnip as a substantial source of diverse bioactive compounds. However, detailed investigation on the pure compounds derived from Turnip green, their bioavailability, transport and metabolism after consumption is further needed. Additional studies on their biological activity are crucial to develop dietary recommendations on the effective dosage and dietary recommendation of Turnip greens for nutrition and health.

## Introduction

Brassica, the most important genus of plants in *Cruciferae* (also called *Brassicaceae*) family, consists of about 350 genera and almost 3,500 species [1]. The *Brassica* plants are very rich in several nutritional (carbohydrates, lipids, protein, vitamins, minerals) and phytochemical components (glucosinolates, isothiocyanates, flavonoids, phenolics) of medicinal value [2]. Their roots, stems, leaves, flower buds, sprouts and seeds were historically used for food and as medicine [2–5]. Health benefits of *Brassic*a were often attributed to glucosinolates [6] and phenolic compounds [7], that induce a variety of physiological functions including antioxidant and anti-inflammatory activity, regulate enzymes production and participate in apoptosis and the cell cycle control [2]. *B*. *oleracea* (broccoli, cauliflower, kale, cabbage, Brussels sprouts, and kohlrabi) is the most famous species of genus *Brassica* and due to its worldwide cultivation and high consumption, its nutritional and remedial features have been extensively studied. However, the other members of *Brassicaceae* family (i.e., *B*. *juncea*, *B*. *napus*, *B*. *nigra*, *B*. *carinata* and *B*. *rapa)* that are far less studied, are as well important constituents of human diet and are valuable sources of vegetable oil [3].

*Brassica rapa ssp*. *rapa* or Turnip is one of the most important leaf and root crops worldwide [2]. It is cultivated for its delicious roots and leaves (greens) which are reaped during the vegetative period; while the Turnip tops, fructiferous stems with the flower buds and surrounding leaves, are consumed before opening and while still green [8]. Young Turnip roots are commonly consumed raw in salads, yet, the Turnip greens and tops are usually served cooked or steamed. Turnip leaves are characterized by a bitter taste, which differentiates them from other *Brassica* vegetables such as broccoli, cabbage or cauliflower [2,9]. Because of the trace amount of phenolic compounds and trivial antioxidant capacity, Turnip root is considered to be less beneficial to human health in comparison to Turnip tops and leaves [2]. Although there were a few published articles about *Brassicaceae* family, so far only one review has summarized phytochemical compounds in Turnip roots, leaves and tops [2]. However, that review was not a systematic review, did not report the quantity of phytochemicals identified in Turnip, and did not focus on Turnip greens (leaves), which is considered to be the most promising health promoting part of the plant. To help fill this literature gap, we performed the present systematic review focusing on Turnip greens, we searched the literature systematically and evaluated both the profile and quantity of phytochemicals commonly found in Turnip greens in order to provide a comprehensive overview of the current literature and provide insights for future research.

## Methods

### Literature search, study selection criteria and data extraction

This review was conducted following a pre-defined protocol and recently published guideline on how to perform systematic reviews [10] and in accordance with the PRISMA [11] guidelines. Four bibliographic databases (PubMed, Embase, Web-of-Science and Cochrane Central Register of Controlled Trials) were searched to identify published studies until April 8^th^, 2020 (date last searched) that examined the nutrient and bioactive composition of turnip greens. The search terms we used were related to nutrient and bioactive compounds (e.g. nutrients, metabolism, phytochemical, carbohydrate, fatty acids) and the plant (Turnip green, *Brassica rapa ssp*. *rapa*) (**S1 Table in S1 File**). We did not apply any restrictions on language and date. The conference abstracts, letters to the editor, book chapters, and editorials were not included in current review. We additionally screened the reference lists of the included studies to retrieve further relevant publications.

Studies were included if they met the following inclusion criteria: (i) used samples of Turnip greens (the leaves); and (ii) evaluated nutrients and bioactive compounds. Studies focusing on other *Brassica rapa* subspecies such as Chinese cabbage *Brassica rapa* ssp *pekinensis* [12] were not included in the review to ensure easier data interpretation. Two reviewers independently evaluated the titles and abstracts according to the selection criteria. For each potentially eligible study, two reviewers assessed the full-texts. In cases of disagreement, the decision was reached by consensus between the two, or in consultation with the third reviewer. Two reviewers independently extracted the data using a predesigned data extraction form, including information on the first author and publication year, name of the phytochemical and its concentration (if reported).

## Results

### Study selection

**Fig 1:**
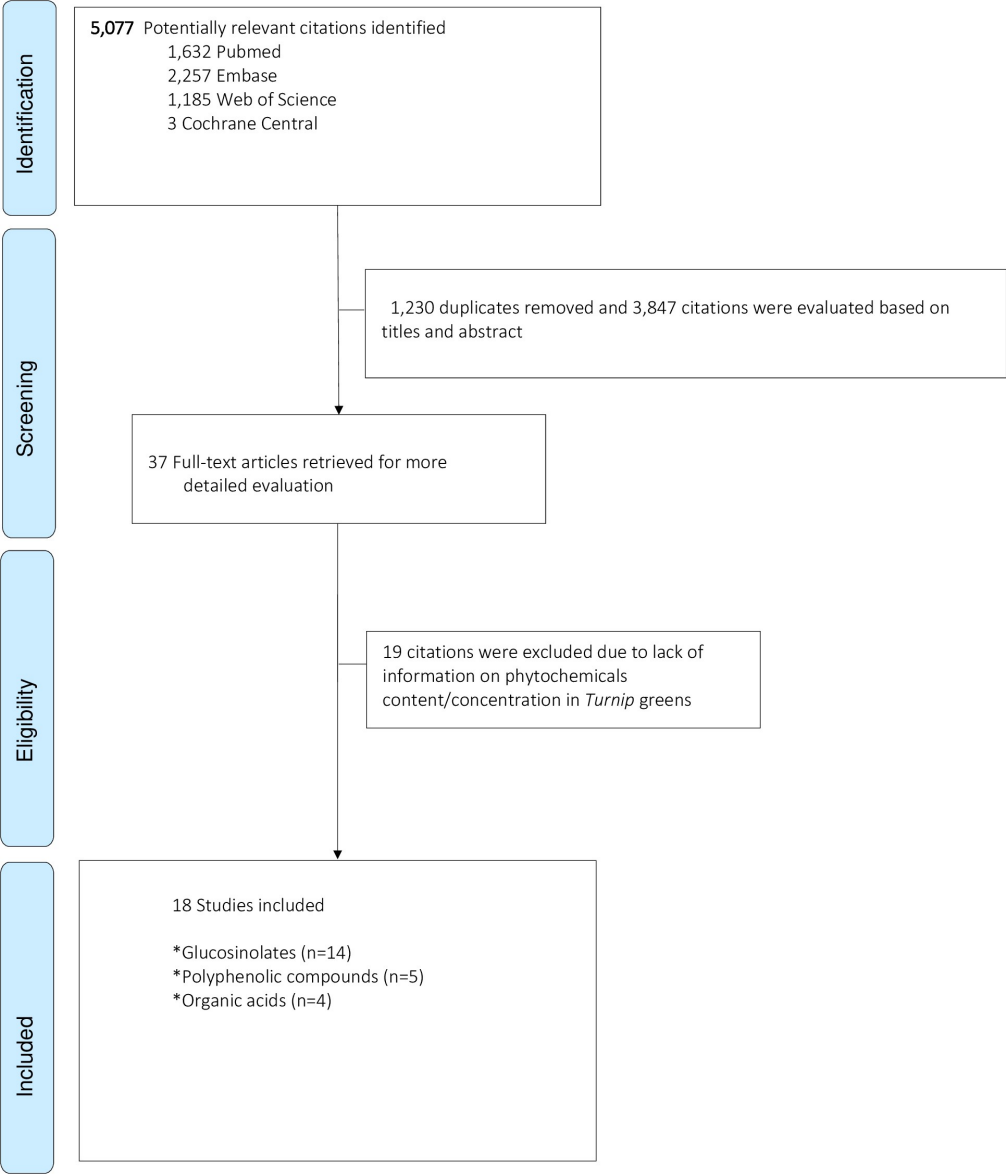
Flowchart of studies included in current review.

**Fig 2:**
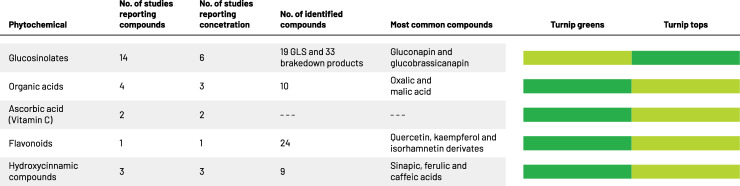
Summary of the most important findings. The shade of green indicates the increasing content of specific phytochemical (darker green color indicates higher phytochemical content in specific anatomical part of the plant); Details can be found in S2 and S3 Tables in S1 File.

Based on the search strategy 5,077 potentially relevant citations were identified and after removing 1,230 duplicates, 3,847 abstracts and titles were evaluated according to inclusion and exclusion criteria. Full texts of 37 studies were evaluated, among which 18 studies were eligible to be included in the current review (**Fig 1**). The majority of included studies (n = 14; 82.4%) focused on glucosinolates and their breakdown products in Turnip greens, four studies reported on organic acids and additional five on phenolic compounds. Among included studies nine studies (50%) provided information on phytochemical’s concentration in Turnip greens. In the 18 included studies for final analysis, there were 129 phytochemicals and metabolites reported in *Turnip* greens. Summary of the most important findings can be found in **Fig 2.**

#### Glucosinolates and breakdown metabolites

Glucosinolates (GLS) are nitrogen- and sulfur-containing plant secondary metabolites that are abundant in the *Brassicaceae* family [13]. Depending on their amino acid precursor, GLSs can be grouped into three chemical classes, aliphatic, aromatic and indole GLS [14]. If cruciferous is consumed raw, GLSs are hydrolyzed in proximal part of the gastrointestinal tract by endogenous plant enzyme myrosinase to several bioactive breakdown metabolites (isothiocyanates, nitriles, thiocyanates, epithionitriles, and oxazolidine) [14]. When vegetables are thermally processed before consumption, plant myrosinase is inactivated and GLS either partially absorbed in stomach of passed to colon where they are hydrolyzed by the intestinal microbime [15]. GLSs are reported to have disease prophylactic and therapeutic effects mediated via their anti-inflammatory, antioxidant, chemopreventive, cytotoxic and anti-cancer activities [2,16].

In current review, nineteen different GLSs were reported across 14 studies. Total GLS content varied from 17.78 to 74 μmol/g dry weight [8,17–20] and aliphatic glucosinolate were the predominant GLSs (14 aliphatic, 4 indol and 1 aromatic). In particular, gluconapin was the most common GLS in five studies, followed by glucobrassicanapin; while in study by Vieites-Outes et al. napin was the major compound in all samples followed by goitrin [21]. Thirty-three GLS breakdown products were identified, among them 19 isothiocyanates, 10 nitriles, and 4 epithionitriles (**Table 1, S2 Table in S1 File**). Epithionitriles were the predominant breakdown products due to the high abundance of alkenyl GLSs.

**Table 1:** Glucosinolates and their breakdown products in Turnip greens.

No.	Compound name	Compound concentration	Study (Lead author, year of publication)
**Aliphatic Glucosinolates**			
1	4-(methylsulfanyl)butyl (Glucoraphanin)	0.007–0.35 μmol/g dw	Klopsch et al, 2018 [22] Padilla et al, 2007 [18]; Francisco et al, 2011 [8] Francisco et al, 2009 [19]; Lee et al, 2013 [23]
2	4-Methylthiobutyl (Glucoerucin)	0.005 μmol/g dw	Padilla et al, 2007 [18]; Bonnema et, 2019 [24]; Yang et al, 2010 [25]; Klopsch et al, 2017 [26]
3	2-hydroxy-4-pentenyl/Gluconapoleiferin	0.15–0.3 μmol/g dw	Klopsch et al, 2018 [22] Padilla et al, 2007 [18]; Bonnema et, 2019 [24]; Yang et al, 2010 [25]; Klopsch et al, 2017 [26]
4	2-(R)-2-hydroxy-3-butenyl (Progoitrin)	0.32–0.8 μmol/g dw	Klopsch et al, 2018 [22] Padilla et al, 2007 [18]; Francisco et al, 2011 [8] Francisco et al, 2009 [19];Cartea et al, 2012 [17], Lee et al, 2013 [23]; Bonnema et, 2019 [24]; Yang et al, 2010 [25]; Francisco et al, 2010 [27]
5	4-pentenyl / Glucobrassicanapin	0.69–22.16 μmol/g dw	Klopsch et al, 2018 [22] Padilla et al, 2007 [18]; Francisco et al, 2011 [8];Cartea et al, 2012 [17]; Lee et al, 2013 [23]; Bonnema et, 2019 [24]; Yang et al, 2010 [25]
12	5-(methylsulfanyl)pentyl (Glucoalyssin)	1.2 μmol/g dw	Klopsch et al, 2018 [22] Padilla et al, 2007 [18]; Klopsch et al, 2017 [26]
11	3-(methylsulfinyl)propyl (Glucoiberin)	1.59 μmol/g dw	Klopsch et al, 2018 [22] Padilla et al, 2007 [18]
6	3-butenyl(Gluconapin)	2.04–26.93 μmol/g dw	Klopsch et al, 2018 [22]; Padilla et al, 2007 [18]; Bonnema et, 2019 [24]; Yang et al, 2010 [25]; Francisco et al, 2010 [27]
7	1-methylpropyl (Glucocochlearin))	n.a.	Klopsch et al, 2017 [26]
8	3-Methylthiopropyl (Glucoiberverin)	n.a.	Padilla et al, 2007 [18]
9	2-(S)-2-Hydroxy-3-butenyl/ Epiprogoitrin	n.a.	Padilla et al, 2007 [18]
10	2-propenyl (Sinigrin)	n.a.	Klopsch et al, 2018 [22]
13	5-Methylthiopentyl (Glucoberteroin)	n.a.	Bonnema et, 2019 [24]; Yang et al, 2010 [25]; Klopsch et al, 2017 [26]
14	4-(methylsulfinyl)butyl	n.a.	Klopsch et al, 2018 [22]
**Indolyl Glucosinolates**			
15	1-methoxy-3-indolylmethyl/Neoglucobrassicin	0.04–1.06 μmol/g dw	Klopsch et al, 2018 [22] Padilla et al, 2007 [18]; Francisco et al, 2011 [8] Francisco et al, 2009 [19];Cartea et al, 2012 [17]; Liang et al (b), 2006 [28]
16	3-indolylmethyl (Glucobrassicin)	0.03–1.71 μmol/g dw	Klopsch et al, 2018 [22] Padilla et al, 2007 [18]; Lee et al, 2013 [23]
17	4-hydroxy-3-indolylmethyl/4-Hydroxyglucobrassicin	0.04–0.98 μmol/g dw	Klopsch et al, 2018 [22] Padilla et al, 2007 [18]; Francisco et al, 2011 [8] Francisco et al, 2009 [19];Cartea et al, 2012 [17]
18	4-methoxy-3-indolylmethyl/4-Methoxyglucobrassicin	0.01 μmol/g dw	Klopsch et al, 2018 [22] Padilla et al, 2007 [18]
**Aromatic Glucosinolates**			
19	2-phenylethyl (Gluconasturtiin)	0.09–2.68 μmol/g dw	Klopsch et al, 2018 [22] Padilla et al, 2007 [18], Francisco et al, 2009 [19];Cartea et al, 2012 [17]; Lee et al, 2013 [23]; Yang et al, 2010 [25]
**Isothiocyanate (ITC)**			
20	Goitrin	1.32–2.55 μmol/g dw	Vieites-Outes et al, 2016 [21]
21	Napin	0.84–1.06 μmol/g dw	Vieites-Outes et al, 2016 [21]
22	sec-butyl ITC	n.a.	Klopsch et al, 2018 [22]; Afsharypuor S. et al, 2010 [29]
23	1-isothiocyanato-4-(methylthio)butane (Erucin)	n.a.	Vieites-Outes et al, 2016 [21]
24	3-butenyl ITC	n.a.	Klopsch et al, 2018 [22]; Afsharypuor S. et al, 2010 [29]
25	5-vinyl-1,3-oxazolidine-2-thione	n.a.	Klopsch et al, 2018 [22]
26	4-pentenyl ITC	n.a.	Klopsch et al, 2018 [22]; Afsharypuor S. et al, 2010 [29]
27	4-(methylsulfanyl)butyl ITC	n.a.	Klopsch et al, 2018 [22]; Afsharypuor S. et al, 2010 [29]
28	5-(methylsulfanyl)pentyl ITC	n.a.	Klopsch et al, 2018 [22]
29	4-(methylsulfinyl)butyl ITC	n.a.	Klopsch et al, 2018 [22]
30	2-phenylethylITC	n.a.	Klopsch et al, 2018 [22]; Afsharypuor S. et al, 2010 [29]
31	phenethylITC	n.a.	Vieites-Outes et al, 2016 [21]
32	2- hexenal	n.a.	Afsharypuor S. et al, 2010 [29]
33	(E)-β -ionone	n.a.	Aharypuor S. et al, 2010 [29]
34	1-methoxyindole-3-carbinol	n.a.	Klopsch et al, 2018 [22]
35	benzyl- ITC	n.a.	Vieites-Outes et al, 2016 [21]
36	allyl-iITC	n.a.	Vieites-Outes et al, 2016 [21]
37	Iberin	n.a.	Vieites-Outes et al, 2016 [21]
38	Sulforaphane	n.a.	Vieites-Outes et al, 2016 [21]
**Nitriles**			
39	3-methylpentanenitrile	n.a.	Klopsch et al, 2018 [22]
40	4-pentenenitrile	n.a.	Klopsch et al, 2018 [22]
41	3-hydroxypentenenitrile	n.a.	Klopsch et al, 2018 [22]
42	5-hexenenitrile	n.a.	Klopsch et al, 2018 [22]
43	5-(methylsulfanyl)pentanenitrile	n.a.	Klopsch et al, 2018 [22]
44	6-(methylsulfanyl)hexanenitrile	n.a.	Klopsch et al, 2018 [22]
45	5-(methylsulfinyl)-pentanenitrile	n.a.	Klopsch et al, 2018 [22]
46	3-phenylpropanenitrile	n.a.	Klopsch et al, 2018 [22]
47	indole-3-acetonitrile	n.a.	Klopsch et al, 2018 [22]
48	1-methoxyindole-3-acetonitrile	n.a.	Klopsch et al, 2018 [22]
**Epithionitrile**			
49	4,5-epithiopentanenitrile	n.a.	Klopsch et al, 2018 [22]
50	3-hydroxy-4,5-epithiopentanenitrile	n.a.	Klopsch et al, 2018 [22]
51	5,6-epithiohexanenitrile	n.a.	Klopsch et al, 2018 [22]
52	3-hydroxy-5,6-epithiohexanenitrile	n.a.	Klopsch et al, 2018 [22]

Compound concentrations across studies: In current table upper detection, range is presented. More details on chemicals concentrations in each study can be found in **S3 Table in S1 File**.

The evidence on variations in GLS content among the Turnip anatomical parts was inconsistent. Cartea et al, reported total GLS as 30.74 μmol/g and 19.50 μmol/g dry weight for Turnip greens and Turnip tops, respectively. One study reported similar content [27], while in two studies total GLSs were more abundant in Turnip tops [8,19]. In addition, 3-Butenyl GLS was predominant in both Turnip top and greens, while, in tubers 2-hydroxy-3-butenyl GLS and 2-phenylethyl GLS was found in high amounts. Epithionitriles were the major hydrolysis products with 4,5-epithiopentanenitrile and 3-hydroxy-4,5-epithiopentanenitrile being the principal compounds [20]. Consistent with the previous results, Bonnema et al reported substantial differences in GLS profiles between aboveground tissues and Turnip tuber, reflecting the differences in their physiological role. In particular, glucoerucin and glucoberteroin were found in considerably high amounts in tubers, but were barely detectable in leaves; the gluconeobrassicin in contrary, was more abundant in Turnip leaves [24].

The nutritional content of Turnip, besides varying among the anatomical structures and varieties, was affected by cooking preparations. In a study done by Vieites-Outes et al, steaming resulted in an increase (+17%) of the amount of total GLS, and while boiling resulted in a decrease (–50%) in the amount of total GLS [21]. Similarly, in study by Francisco et al., steaming was the method that better preserved GLS and phenolic compounds while conventional boiling and high-pressure cooking methods presented similar rate of losses of total GLS content (64%) [27]. Nevertheless, genotype, environment and the stage of development of the plant were important factors to affect the GLSs content. In particular, Francisco et al. found genotype largely influences the aliphatic glucosinolates in the plant, while the indolic glucosinolate content was affected by both the genotype and its interaction with the environment [8].

The GLSs the breakdown products, isothiocyanates and indoles, highly reactive and potent inducers of Phase II enzymes [30], have been associated with diverse health-promoting effects [2]. In current review, sulforaphane, a potent anti-cancer isothiocyanate [18], was identified in a single study and in small quantities in the fresh *Turnip* sample 21]. Its precursors, glucoraphanin, was detected in a few Turnip varieties, was present in considerably smaller amounts (ranging from 0.003–0.35 μmol/g dry weight); whoever, the role of this aliphatic GLS in human health shall merits to be explored further due to its potential to convert to sulforaphane [8,18,19,22,23]. On the other hand, progoitrin (found in cauliflower, cabbage, mustard, turnip, raddish, bamboo shoot and cassava), is considered an anti-nutritional GLS (due to potential anti-thyroid effects) [31]. In current review, progoitrin, was present in most Turnip varieties in low concentration (0.32 to 1.5 μmol g^1^ dw) [8,9,17,18,22]. Although, the evidence on potential goitrogenic effects of this GLS comes from animals the consumption of vegetables containing progoitrin should be carefully monitored in people with thyroid diseases.

#### Organic acids

Organic acids are intermediates of major carbon metabolism in plant cells and are involved in various biochemical pathways (glycolysis, photorespiration, the glyoxylate cycle) and play an important role in controlling plant cell physiology. Organic acids have been also implicated to control biochemical and physiological processes *in vivo* and are known to have antioxidant activity [32].

Four studies [24,27,33,34] reported ten organic acid in turnip greens leaves aconitic, citric, ketoglutaric, malic, shikimic, fumaric, oxalic, ascorbic, succinic and glutamic acids (**Table 2, S3 Table in S1 File**). Arias-Carmona et al, determined the organic acids in 44 samples of *Brassica rapa var*. *rapa L*. greens and tops and all samples presented a profile composed of at least four organic acids: citric, malic, oxalic, and ascorbic acids [34]. The oxalic acid content was the highest in the analyzed samples, and varied between 138.40 and 83.89 mg/100 g fresh weight for Turnip greens and turnip tops respectively. The malic acid was second most concentrated and its content in the product varied between 89.34 mg/100 g fresh weight in Turnip greens and 37.12 mg/100 g fresh weight in Turnip tops. Citric and ascorbic acids contents in Turnip greens were 56.75 and 37.13 mg/100 g fresh weight respectively, and in contrast, the content of acids was higher in Turnip tops in comparison to Turnip greens [34]. In study by Fernandes et al, the vitamin C content in fresh Turnip greens and tops was 62 mg/100g fresh weight and 46 mg/100g fresh weight, respectively. Also, the concentration of vitamin C was dramatically reduced by the processing method; after steaming treatment, the loss was 64% with respect to untreated fresh material and after high pressure and conventional boiling, vitamin C was not found in the edible parts [27]. In the study by Fernandes et al. citric, ketoglutaric, malic, aconitic, shikimic and fumaric acids were detected in all edible Turnip organs, however, their content varied with higher content being present in flower buds, leaves and stems in comparison to the roots [33]. Malic acid was the major compound in those edible parts with roots exhibiting significantly higher amount (ca. 81%), followed by leaves and stems (ca. 65%) while flower buds showed a significantly lower content (ca. 44%), suggesting that malic acid content may be useful to differentiate *Turnip* edible parts [33]. Liang et al. in two publications [28] detected malic, succinic and glutamic acid without providing information on specific acid concentrations. Trans/cis-hydroxycinnamates of malic acids were also reported in turnip leaves extracts: sinapoylmalate, feruloylmalate and coumaroylmalate, and their levels were higher levels in methyl ester of jasmonic acid than in jasmonic acid itself. In addition, after treatment with methyl ester of jasmonic acid after indole 3-acetic acid (an important plant hormone controlling a variety of developmental processes) was also increased.

**Table 2:** Polyphenolic compounds identified in Turnip greens and their concentrations.

Flavonols			
No	Compound name	Compound concentration	Study (Lead author, year of publication)
1	quercetin-3-O-sophoroside	0.02 μmol/g dw	Francisco et al, 2009 [19]
2	quercetin-3-O-(feruloyl)sophoroside	0.3 μmol/g dw	Francisco et al, 2009 [19]
3	quercetin-3-O-sophoroside-7-O-glucoside	0.4 μmol/g dw	Francisco et al, 2009 [19]
4	kaempferol-3-O-sophoroside-7-O-glucoside with methoxycaffeoyl	0.40 μmol/g dw	Francisco et al, 2009 [19]
5	kaempferol-3-O-sophoroside-7-O-glucoside	2.05 μmol/g dw	Francisco et al, 2009 [19]
6	quercetin-3-O-(sinapoyl)-sophoroside-7-O-glucoside	2.63 μmol/g dw	Francisco et al, 2009 [19]
7	kaempferol-3-Otriglucoside-7-O-glucoside	n.a	Francisco et al, 2009 [19]
8	quercetin-3,7-di-O-glucoside	n.a	Francisco et al, 2009 [19]
9	kaempferol3,7-di-O-glucoside	n.a	Francisco et al, 2009 [19]
10	isorhamnetin-3,7-di-O-glucoside	n.a	Francisco et al, 2009 [19]
11	kaempferol-3-O-diglucoside	n.a	Francisco et al, 2009 [19]
12	kaempferol-3-O-sophoroside	n.a	Francisco et al, 2009 [19]
13	quercetin-7-O-glucoside	n.a	Francisco et al, 2009 [19]
14	kaempferol-7-O-glucoside	n.a	Francisco et al, 2009 [19]
15	isorhamnetin-7-O-glucoside	n.a	Francisco et al, 2009 [19]
16	1- methoxycaffeoyl	n.a	Francisco et al, 2009 [19]
17	1-caffeoyl	n.a	Francisco et al, 2009 [19]
18	1-sinapoyl	n.a	Francisco et al, 2009 [19]
19	1-feruloyl	n.a	Francisco et al, 2009 [19]
20	3-p-coumaroyl	n.a	Francisco et al, 2009 [19]
21	3-caffeoyl	n.a	Francisco et al, 2009 [19]
22	3-sinapoyl	n.a	Francisco et al, 2009 [19]
23	7-feruloyl	n.a	Francisco et al, 2009 [19]
24	1-p-coumaroyl	n.a	Francisco et al, 2009 [19]
**Phenolics**			
25	3-caffeoylquinic acid	0.75 μmol/g dw	Lin et al, 2010 [35]
26	kaempferol-3-O-(methoxycaffeoyl)sophoroside-7-O-glucoside	2.60 μmol/g dw	Francisco et al, 2009 [19]
27	kaempferol 3-O-(feruloyl/caffeoyl)-sophoroside-7-O-glucoside	3.99 μmol/g dw	Fernandes at al, 2007 [33]
28	kaempferol 3-O-sophoroside-7-O-glucoside	n.a	Fernandes at al, 2007 [33]
29	kaempferol 3-O-sophoroside-7-O-sophoroside	n.a	Fernandes at al, 2007 [33]
30	kaempferol 3,7-O-diglucoside, isorhamnetin 3,7-O-diglucoside	n.a	Fernandes at al, 2007 [33]
31	kaempferol 3-O-sophoroside, 1,2-disinapoylgentiobiose	n.a	Fernandes at al, 2007 [33]
32	1,20-disinapoyl-2-feruloylgentiobiose, kaempferol 3-O-glucoside	n.a	Fernandes at al, 2007[33]
33	isorhamnetin 3-O-glucoside	n.a	Fernandes at al, 2007 [33]
34	3-O-caffeoyldiglucoside	n.a	Lin et al, 2010 [35]
35	3-O-diglucoside	n.a	Lin et al, 2010 [35]
36	3-O-feruloyl diglucoside	n.a	Lin et al, 2010 [35]
37	3-O-feruloyldiglucoside-7-O-glucoside	n.a	Lin et al, 2010 [35]
38	3-O-glucoside	n.a	Lin et al, 2010 [35]
39	3-O-glucoside-7-O-glucoside	n.a	Lin et al, 2010 [35]
40	3-O-hydroxyferuloyldiglucoside	n.a	Lin et al, 2010 [35]
41	3-O-hydroxyferuloyldiglucoside-7-O-glucoside	n.a	Lin et al, 2010 [35]
42	3-O-p-coumaroyldiglucoside-7-O-glucoside	n.a	Lin et al, 2010 [35]
43	3-O-p-coumaroyldiglucosidee	n.a	Lin et al, 2010 [35]
44	3-O-sinapoyldiglucoside	n.a	Lin et al, 2010 [35]
45	3-O-sinapoylldiglucoside-7-O-glucoside	n.a	Lin et al, 2010 [35]
46	4-p-coumaroylquinic acid	n.a	Lin et al, 2010 [35]
47	5-feruloylquinic acidd	n.a	Lin et al, 2010 [35]
48	5-p-coumaroylquinic acid	n.a	Lin et al, 2010 [35]
49	kaempferol 7-O-glucoside	n.a	Lin et al, 2010 [35]
50	kaempferol dihexoside	n.a	Lin et al, 2010 [35]
**Hydroxycinnamic acids**			
51	1,2,2`-trisinapoylgentiobioside	0.39 μmol/g dw	Francisco et al, 2009 [19]
52	3-caffeoyl quinic acid	0.75 μmol/g dw	Francisco et al, 2009 [19]
53	1,2-disinapoylgentiobioside	1.43 μmol/g dw	Francisco et al, 2009 [19]
54	sinapic acid derivates	12.46 μmol/g dw	Francisco et al, 2009 [19]; Fernandes at al, 2007 [33]
55	1-sinapoyl-2-feruloylgentiobioside	3.19 μmol/g dw	Francisco et al, 2009 [19]
56	3-p-coumaroylquinin acid	3.41 μmol/g dw	Francisco et al, 2009 [19]
57	caffeic acid	n.a	Francisco et al, 2009 [19]
58	ferulic acid derivates	n.a	Fernandes at al, 2007 [33]
59	3-p-coumaroylquinic acid	n.a	Lin et al, 2010 [35]
**Organic acids**			
60	Shikimic acid*	1.9–30.33g/100g dw	Fernandes at al, 2007 [33]
61	Oxalic acid	138.40 mg/100g dw	Arias-Carmona et al [34]
62	Aconitic acid *	16.08–1247.2	Fernandes at al, 2007 [33]
63	Ascorbic acid	37.13–62 mg/100g dw	Arias-Carmona et al [34]
64	Fumaric acid*	39.13–168.35 mg/100g dw	Fernandes at al, 2007 [33]
65	Citric acid	56.75 mg/100g dw	Fernandes at al, 2007 [33]; Arias-Carmona et al [34]
66	Malic acid	89.34 mg/100g dw	Fernandes at al, 2007 [33]; Liang et al, 2006 [28]; Arias-Carmona et al [34]
67	Succinic acid	n.a.	Liang et al, 2006 [28]
68	Glutamic acid	n.a.	Liang et al, 2006 [28]
69	Ketoglutaric	n.a.	Fernandes at al, 2007 [33]
70	Trans-sinapoylmalate	n.a.	Liang et al, 2006 [28]
71	Trans-feruloylmalate	n.a.	Liang et al, 2006 [28]
72	Trans-coumaroylmalate	n.a.	Liang et al, 2006 [28]
73	Cis-sinapoylmalate	n.a.	Liang et al, 2006 [28]
74	Cis-feruloylmalate	n.a.	Liang et al, 2006 [28]
75	Cis-coumaroylmalate	n.a.	Liang et al, 2006 [28]
76	5-Hydroxyferuloyl malate	n.a.	Liang et al, 2005 [36]
**Plant hormone**			
77	Indole 3-acetic acid	n.a.	Liang et al, 2006 [28]

*content reported in leaves and stems.

#### Polyphenolic compounds

*Phenolic compounds* is a generic term, which refers to more than 8,000 secondary metabolites in plants, which are categorized into different classes depending on their structure and subcategorized within each class according to the number and position of hydroxyl group and the presence of other substituents [19]. They are classified into flavonoids (flavonols, flavones, flavan-3-ols, anthocyanidins, flavanones, isoflavones and others) and non-flavonoids (phenolic acids, hydroxycinnamates, stilbenes and others); with flavonoids and hydroxycinnamic acid derivates being the most diverse compounds [37]. They play a role in protection against UV, pigmentation, stimulation of nitrogen-fixing nodules and are important biologically active constituents of the human diet [38]. In particular, they have strong antioxidant and free radical-scavenging activities and may regulate the expression of various genes encoding important metabolic enzymes and thus are involved in important physiological processes [39]. Phenolic compounds also interact with human microbiome in the lower gastrointestinal tract, where they can positively influence the composition and activity of the microbiota, which ultimately leads to overall better health in humans [40,41].

We have identified five studies [19,28,33–35] reporting polyphenolic components in Turnip greens. Overall, 59 polyphenolic compounds (phenols, flavonoids, flavonol glycosides) were identified and listed in **Table 2 and S3 Table in S1 File.**

Francisco et al. reported more than 30 phenolic compounds in *Turnip* greens and tops. The main naturally occurring flavonoids identified were kaempferol, quercetin, and isorhamnetin glycosylated and acylated with different hydroxycinnamic acids [19]. Total flavonoids content of *Turnip* greens and tops were similar, 29.7 μmol/g dry weight and 28.44 μmol/g dry weight, respectively [19]. The isorhamnetin was one of the major flavonoids in *Turnip* greens that is not present in the *B*. *oleracea* family, and serve as a biochemical marker of *Turnip* varieties. *Turnip* green was also shown to contain 22.8 μmol/g dry weight of hydroxycinnamic compounds compared to 10.2 μmol/g dry weight in *Turnip* top [19]. Sinapic acid was the major hydroxycinnamic acid and the main phenolic compound in turnip greens [19]. Its concentration in *Turnip* greens was considerably higher than in *Turnip* tops, 12.46 mol/g dry weight and 2.14 mol/g dry weight, respectively [19]. Overall, total phenolic content revealed a higher amount in Turnip greens (31.51 μmol/g dry weight), than in Turnip tops (14.80 μmol/g dry weight). The authors hypothesized this disparity from the high amount of sinapic acid in Turnip greens, which is present in lower quantities in Turnip tops [8]. Furthermore, after cooking, total phenolics content in Turnip greens was reduced in 15%, 75% and 72% in steaming, high-pressure cooking, and conventional boiling, respectively [8].

Fernandes at al. showed that among identified phenolic compounds, kaempferol 3-O-sophoroside-7-O-glucoside, kaempferol 3-O-(feruloyl/caffeoyl)-sophoroside-7-O-glucoside, isorhamnetin 3,7-O-diglucoside and isorhamnetin 3-O-glucoside as the main phenolics, were present in highest amounts [33]. The group reported high organic acid content ranging from 36 to 51 g/kg dry weight. Aconitic, citric, ketoglutaric, malic, shikimic and fumaric acids were detected in all edible parts, but there were some qualitative differences, and a higher content of these compounds in flower buds,leaves and stems than in the roots were observed [33].

Lin et al, compared the similarity of the phenolic components of 17 leafy vegetables from Brassica species other than Brassica oleracea [35]. Among those, twelve plants were divided into three groups that had similar chromatographic patterns; while the remaining five vegetables namely gai choy, baby napa, rapini, baby Shanghai bok choy, and napa had individual phenolic compounds patterns [35]. Turnip greens were grouped together with yu choy, and yu choy tip in group and peak 5-caffeoylquinic acid distinguished this group from the other two groups (group I: baby gai choy, baby mustard greens, and mustard greens with peak 4-p-coumaroylquinic acid and group III: baby bok choy, bok choy, bok choy sum, bok choy tip, Shanghai bok choy, and Taiwan bok choy with peak caffeic, ferulic and sinapic acid glucosides) [35].

### Literature gaps and directions for future research

Higher intake of cruciferous vegetables was associated with multiple health benefits and lower all-cause and cardiovascular mortality [42–46]. Those health benefits were attributed to their high GLS content, phenolic derivatives and especially flavonoids and hydroxycinnamic acids content [47–49]. Previous human and clinical studies, however, explored the health benefits of cruciferous vegetables as a group, while studies focusing specifically on Turnip or Turnip greens in humans remain scarce. Conversely, emerging in vitro and in vivo studies are supporting the role of Turnip in improving health via its antioxidant and anti-inflammatory properties [50–52].

Based on current data, it is difficult to speculate which anatomical part of Turnip may have the most promising antioxidant capacity. The flower buds of Turnip exhibited the strongest antioxidant capacity compared with other edible parts in the 1,1-diphenyl-2-picrylhydrazine (DPPH) radical scavenging assay. Flower buds were shown with an IC25 value (extract concentration providing 25% scavenging activity) of 0.47 mg/mL, followed by leaves and stems (IC25 = 0.56 mg/mL), while the roots showed the lowest antioxidant capacity with an IC25 value of 1.44 mg/mL [33]. In contrary, another study reported best antioxidant potency of aqueous Turnip roots whose DPPH radical scavenging activity was equivalent to vitamin C; followed by Turnip greens extract [53]. Those contradicting may be because the antioxidant activity of Turnip was mostly assessed using the crude extracts of Turnip instead of isolated compounds. For example, flavonoids, one of the most abundant phytochemicals in Turnip greens, have been detected in Turnip greens and tops but not in roots, while GLSs are suggested to be more abundant in Turnip roots in comparison to turnip tops and greens.

In addition, Turnip genotype, environmental factors, cooking preparations and the other dietary habits and intestinal microbiome have been suggested to affect the bioavailability of GLSs, flavonoids and isothiocyanates [54] and may subsequently influence the antioxidant capacity of Turnip in vivo. For example, GLSs are relatively stable in plant cells, but cell damage by cutting, chopping or chewing food relieves myrosinase (β-thioglucosidase) which hydrolyzes GLSs and produces molecules of β-d-glucose and an unstable aglycone called thiohydroximate-O-sulfonate, which spontaneous reorganization results in the release of sulfate ion and the formation of plants metabolites [54]. Previous studies have suggested that the inactivation of myrosinase, the processing and storage conditions, and the association with other food constituents are essential factors that affect GLS absorption and their metabolism.

It is, therefore, necessary to better understand the metabolism of the major health-promoting phytochemicals in Turnip but also their breakdown products and to define sensitive biomarkers of Turnip intake. When exploring health benefits of Turnip or any other cruciferous in humans, usual dietary patterns should be carefully taken into consideration rather than focusing solely on metabolism of individual molecules (e.g. food components may interact with absorption and metabolism) [55]. Furthermore, dose-response curves should be explored for potential therapeutic and adverse effects associated with the consumption of phenolics, GLSs and their breakdown products (e.g. with progoitrin).

## Conclusions

This review identified 129 phytochemicals reported in Turnip greens. The major active constituents of Turnip greens were glucosinolates, isothiocyanates, flavonoids, and phenylpropanoids. Flavonoids, mainly present as quercetin, kaempferol, and isorhamnetin derivatives, were detected in Turnip greens but not in roots emphasizing the need for eating the greens rather than its conventional consumption pattern. Emerging evidence has suggested a beneficial role of Turnip consumption to human’s health and promoting the Turnip green as an considerable source of health-protective compounds. However, further research and investigation on pure compounds or extracts derived from Turnip greens, their bioavailability, transport and metabolism after consumption would help further understand their potential benefits to human health and underlying mechanisms of action. Furthermore, observational and interventional studies exploring their biological activity and associated health benefits down the road would help develop relevant dietary recommendations and/or guidelines regarding adequate consumption and associated health benefits of Turnip greens.

## Supporting information

S1 File Search strategy and additional files. (DOCX)

S2 File PRISMA checklist. (DOC)
