# Supplementary material for: Phytochemical characterization of turnip greens (Brassica rapa ssp. rapa): A systematic review
Source: PLoS One. 2021 Feb 17;16(2):e0247032. doi: 10.1371/journal.pone.0247032 (PMC7888597; doi:10.1371/journal.pone.0247032)
Supplement: S1 File — (DOCX) [file pone.0247032.s001.docx]

**Online Supplemental Material**

| **S1 Table.** Search strategy |
| --- |
| April 8th, 2020 (last date searched) |
| **Embase.com**  ('metabolism'/exp OR 'metabolomics'/exp OR 'energy metabolism'/exp OR 'metabolic activity assay'/de OR 'phytochemistry'/de OR 'phytochemical'/exp OR 'nutrient content'/de OR 'nutrient'/exp OR 'nutraceutical'/exp OR 'diet supplementation'/de OR 'pharmacokinetics'/exp OR (metabol* OR cometabol* OR co-metabol* OR nutrient* OR nutraceut* OR phytochem* OR phytopharmaceut* OR pharmacokinetic* OR ((dietary OR nutritional) NEAR/3 (supplement*))):ab,ti) OR ('alkaloid'/exp OR 'amine'/exp OR 'amino acid'/exp OR 'carbohydrate'/exp OR 'carotenoid'/exp OR 'disaccharide'/exp OR 'fatty acid'/exp OR 'flavonoid'/exp OR 'indole derivative'/exp OR 'lignan'/exp OR 'lipid'/exp OR 'monosaccharide'/exp OR 'peptide'/exp OR 'polysaccharide'/exp OR 'protein'/exp OR 'purine derivative'/exp OR 'pyrimidine derivative'/exp OR 'stilbene derivative'/de OR 'sugar alcohol'/exp OR 'terpene'/de OR 'amide'/de OR 'avenanthramide'/exp OR ("Amino acid*" OR "Cyanogenic glucoside*" OR "Fatty acid*" OR "Nucleic acid base*" OR "Organic acid*" OR "Organosulfur compound*" OR "Phenolic acid*" OR "Sugar alcohol*" OR Alkaloid* OR Alkane* OR Amine* OR Benzenoid* OR Carotenoid* OR Chlorophyll* OR Disaccharide* OR Flavonoid* OR Indole* OR Lignan* OR Monosaccharide* OR Peptide* OR Polyacetylene* OR Polysaccharide* OR Protein* OR Purine* OR Pyrimidine* OR Stilbene* OR Terpene* OR Terpenoid* OR avenanthramid* OR amide*):ab,ti) AND ('plant extract'/de OR 'plant medicinal product'/de OR 'nutrient content'/de OR 'chromatography'/exp OR 'chemistry'/exp OR (chromatogra* OR electrochromatogra* OR ((plant OR plants) NEAR/3 (extract* OR biochem* OR preparation* OR medicinal*))):ab,ti) AND ('brassica rapa'/de OR (turnip* OR rutabaga* OR rapifera OR septiceps OR ((brassica) NEAR/5 (rapa OR napus))):ab,ti) NOT ([Conference Abstract]/lim OR [Letter]/lim OR [Note]/lim OR [Editorial]/lim) |
| **Web of Science (Core collection)**  TS=(((metabol* OR cometabol* OR co-metabol* OR nutrient* OR nutraceut* OR phytochem* OR phytopharmaceut* OR pharmacokinetic* OR ((dietary OR nutritional) NEAR/2 (supplement*)) OR "Amino acid*" OR "Cyanogenic glucosid*" OR "Fatty acid*" OR "Nucleic acid base*" OR "Organic acid*" OR "Organosulfur compound*" OR "Phenolic acid*" OR "Sugar alcohol*" OR Alkaloid* OR Alkane* OR Amine* OR Benzenoid* OR Carotenoid* OR Chlorophyll* OR Disaccharide* OR Flavonoid* OR Indole* OR Lignan* OR Monosaccharide* OR Peptide* OR Polyacetylene* OR Polysaccharide* OR Protein* OR Purine* OR Pyrimidine* OR Stilbene* OR Terpene* OR Terpenoid* OR avenanthramid* OR amide*) AND (chromatogra* OR electrochromatogra* OR ((plant OR plants) NEAR/3 (extract* OR biochem* OR preparation* OR medicinal*)))) AND (turnip* OR rutabaga* OR rapifera OR septiceps OR ((brassica) NEAR/4 (rapa OR napus)))) AND DT=(article) |
| **Cochrane**  (metabol* OR cometabol* OR co-metabol* OR nutrient* OR nutraceut* OR phytochem* OR phytopharmaceut* OR pharmacokinetic* OR ((dietary OR nutritional) NEAR/3 (supplement*)) OR "Amino acids" OR "Amino acid" OR "Cyanogenic glucoside" OR "Cyanogenic glucosides" OR "Fatty acids" OR "Fatty acid" OR "Nucleic acid base" OR "Nucleic acid bases" OR "Nucleic acid based" OR "Organic acid" OR "Organic acids" OR "Organosulfur compound" OR "Organosulfur compounds" OR "Phenolic acids" OR "Phenolic acid" OR "Sugar alcohol" OR "Sugar alcohols" OR Alkaloid* OR Alkane* OR Amine* OR Benzenoid* OR Carotenoid* OR Chlorophyll* OR Disaccharide* OR Flavonoid* OR Indole* OR Lignan* OR Monosaccharide* OR Peptide* OR Polyacetylene* OR Polysaccharide* OR Protein* OR Purine* OR Pyrimidine* OR Stilbene* OR Terpene* OR Terpenoid* OR avenanthramid* OR amide*) AND (chromatogra* OR electrochromatogra* OR ((plant OR plants) NEAR/3 (extract* OR biochem* OR preparation* OR medicinal*))) AND (turnip* OR rutabaga* OR rapifera OR septiceps OR ((brassica) NEAR/5 (rapa OR napus))) |
| **PubMed**  (((("Metabolism"[mh] OR "Metabolomics"[mh] OR "Energy Metabolism"[mh] OR "Phytochemicals"[mh] OR "Nutrients"[mh] OR "Dietary Supplements"[mh] OR "Pharmacokinetics"[mh] OR metabol*[tiab] OR cometabol*[tiab] OR co-metabol*[tiab] OR nutrient*[tiab] OR nutraceut*[tiab] OR phytochem*[tiab] OR phytopharmaceut*[tiab] OR pharmacokinetic*[tiab] OR ((dietary[tiab] OR nutritional[tiab]) AND (supplement*[tiab]))) OR ("Alkaloids"[mh] OR "Amines"[mh] OR "Amino acids"[mh] OR "Carbohydrates"[mh] OR "Carotenoids"[mh] OR "Disaccharides"[mh] OR "Fatty Acids"[mh] OR "Flavonoids"[mh] OR "Indoles"[mh] OR "Lignans"[mh] OR "Lipids"[mh] OR "Monosaccharides"[mh] OR "Peptides"[mh] OR "Polysaccharides"[mh] OR "Proteins"[mh] OR "Purines"[mh] OR "Pyrimidines"[mh] OR "Stilbenes"[mh] OR "Sugar Alcohols"[mh] OR "Terpenes"[mh] OR "Amides"[mh] OR Amino acid*[tiab] OR Cyanogenic glucoside*[tiab] OR Fatty acid*[tiab] OR Nucleic acid base*[tiab] OR Organic acid*[tiab] OR Organosulfur compound*[tiab] OR Phenolic acid*[tiab] OR Sugar alcohol*[tiab] OR Alkaloid*[tiab] OR Alkane*[tiab] OR Amine*[tiab] OR Benzenoid*[tiab] OR Carotenoid*[tiab] OR Chlorophyll*[tiab] OR Disaccharide*[tiab] OR Flavonoid*[tiab] OR Indole*[tiab] OR Lignan*[tiab] OR Monosaccharide*[tiab] OR Peptide*[tiab] OR Polyacetylene*[tiab] OR Polysaccharide*[tiab] OR Protein[tiab] OR Proteins[tiab] OR Purine*[tiab] OR Pyrimidine*[tiab] OR Stilbene*[tiab] OR Terpene*[tiab] OR Terpenoid*[tiab] OR avenanthramide*[tiab] OR amide*[tiab])) AND (("Plant Extracts"[mh] OR "Chromatography, Liquid"[Mesh] OR chromatogra*[tiab] OR electrochromatogra*[tiab] OR ((plant[tiab] OR plants[tiab]) AND (extract*[tiab] OR biochem*[tiab] OR preparation*[tiab] OR medicinal*[tiab]))))) AND (("Brassica rapa"[mh] OR turnip*[tiab] OR rutabaga*[tiab] OR rapifera[tiab] OR septiceps[tiab] OR ((brassica[tiab]) AND (rapa[tiab] OR rapas[tiab] OR napus[tiab]))))) NOT ((letter[pt] OR news[pt] OR comment[pt] OR editorial[pt] OR congress[pt])) |

| **S2 Table** Glucosinolates content in turnip greens and tops | | | | |
| --- | --- | --- | --- | --- |
| **No.** | **Compound** | **Turnip greens, µmol/g dry weight** | **Turnip tops, µmol/ g dry weight** | **Author** |
| 1 | 4-hydroxyglucobrassicin | 0.98 | 1.02 | Francisco et al, 2011^2^ |
|  |  | 0.05 | NA | Klopsch et al, 2017^3^ |
|  |  | 0.04 | NA | Padilla et al, 2007^4^ |
|  |  | 0 | 0.40 | Francisco et al, 2009^5^ |
|  |  | 0.47 | 0.42 | Francisco et al, 2010^6^ |
| 2 | glucobrassicanapin | 1.69 | 1.84 | Cartea et al, 2012^7^ |
|  |  | 1.9 | 2.38 | Francisco et al, 2011^2^ |
|  |  | 0.87 | 2.05 | Francisco et al, 2009^5^ |
|  |  | 0.69 | NA | Klopsch et al, 2017^3^ |
|  |  | 2.6 | NA | Padilla et al, 2007^4^ |
|  |  | 1.44 | 1.31 | Francisco et al, 2010 |
| 3 | glucobrassicin | 0.3 | 0.26 | Cartea et al, 2012^7^ |
|  |  | 1.26 | 1.3 | Francisco et al, 2011^2^ |
|  |  | 1.71 | 1.17 | Francisco et al, 2009^5^ |
|  |  | 1.50 | 1.54 | Francisco et al, 2010^6^ |
|  |  | 0.03 | NA | Klopsch et al, 2017^3^ |
|  |  | 0.21 | NA | Padilla et al, 2007^4^ |
| 4 | gluconapin | 26.93 | 15.67 | Cartea et al, 2012^7^ |
|  |  | 10.21 | 17.39 | Francisco et al, 2011^2^ |
|  |  | 6.27 | 7.25 | Francisco et al, 2010^6^ |
|  |  | 22.16 | 24.33 | Francisco et al, 2009^5^ |
|  |  | 2.04 | NA | Klopsch et al, 2017^3^ |
|  |  | 25.5 | NA | Padilla et al, 2007^4^ |
| 5 | gluconapoleiferin | 0.15 | 0.15 | Cartea et al, 2012^7^ |
|  |  | 0.3 | NA | Klopsch et al, 2017^3^ |
| 6 | gluconasturtiin | 0.65 | 0.33 | Cartea et al, 2012^7^ |
|  |  | 1.52 | 1.43 | Francisco et al, 2011^2^ |
|  |  | 0.87 | NA | Francisco et al, 2009^5^ |
|  |  | 1.23 | 1.21 | Francisco et al, 2010^6^ |
|  |  | 0.09 | NA | Klopsch et al, 2017^3^ |
|  |  | 1.08 | NA | Padilla et al, 2007^4^ |
| 7 | glucoraphanin | 0.22 | 0.35 | Francisco et al, 2011^2^ |
|  |  | 0.35 | NA | Francisco et al, 2009^5^ |
|  |  | 0.007 | NA | Klopsch et al, 2017^3^ |
|  |  | 0.01 | NA | Padilla et al, 2007^4^ |
| 8 | neoglucobrassicin | 0.39 | 0.28 | Cartea et al, 2012^7^ |
|  |  | 1.06 | 0.89 | Francisco et al, 2011^2^ |
|  |  | 0.04 | NA | Klopsch et al, 2017^3^ |
|  |  | 0.30 | NA | Padilla et al, 2007^4^ |
|  |  | 0.52 | 0.22 | Francisco et al, 2009^5^ |
|  |  | 1.24 | 0.18 | Francisco et al, 2010^6^ |
| 9 | progoitrin | 0.32 | 0.81 | Cartea et al, 2012^7^ |
|  |  | 0.8 | 1.28 | Francisco et al, 2011^2^ |
|  |  | 0.35 | 0.91 | Francisco et al, 2009^5^ |
|  |  | 0.42 | NA | Klopsch et al, 2017^3^ |
|  |  | 1.4 | NA | Padilla et al, 2007^4^ |
|  |  | 0.83 | 0.13 | Francisco et al, 2010 |
| 10 | 4-Methoxyglucobrassicin | 0.01 | NA | Klopsch et al, 2017^3^ |
| 11 | Glucoerucin | 0.005 | NA | Klopsch et al, 2017^3^ |
| 12 | Glucoiberin | 1.59 | NA | Padilla et al, 2007^4^ |
| 13 | Glucoiberverin | 0.22 | NA | Padilla et al, 2007^4^ |
| 14 | glucoalyssin | 1.2 | NA | Padilla et al, 2007^4^ |
| 15 | gluconapoleiferin | 0.11 | NA | Padilla et al, 2007^4^ |
| 16 | total aliphatics | 17.78 | 26.02 | Francisco et al, 2011^2^ |
|  |  | 8.55 | 8.69 | Francisco et al, 2010^6^ |
| 17 | total GLS | 26.84 | 29.11 | Francisco et al, 2009^5^ |
|  |  | 30.74 | 19.5 | Cartea et al, 2012^7^ |
|  |  | 17.78 | 26.02 | Francisco et al, 2011^2^ |
|  |  | 12.99 | 12.85 | Francisco et al, 2010^6^ |
|  |  | 74 | NA | Padilla et al, 2007^4^ |
| 18 | total indolics | 3.3 | 3.21 | Francisco et al, 2011^2^ |
|  |  | 1.71 | NA | Francisco et al, 2009^5^ |
|  |  | 3.21 | 3.14 | Francisco et al, 2010^6^ |

| **S3 Table.** Flavonoids concentrations in turnip tops and turnip greens | | | | |
| --- | --- | --- | --- | --- |
| **No** | **Compound** | **Turnip greens, µmol g^1^ dw** | **Turnip tops, µmol g^1^ dw** | **Author** |
| 1 | Total flavonoids | 29.70 | 28.4 | Francisco et al, 2009^5^ |
| 2 | quercetin-3-O-sophoroside-7-O-glucoside (1) | 0.4 | 0.25 | Francisco et al, 2009^5^ |
| 3 | kaempferol-3-O-sophoroside-7-Oglucoside (3) | 2.05 | 1.47 | Francisco et al, 2009^5^ |
| 4 | kaempferol-3-O-(methoxycaffeoyl)sophoroside-7-O-glucoside (15) | 2.6 | 1.47 | Francisco et al, 2009^5^ |
| 5 | kaempferol-3-O-(caffeoyl)sophoroside-7-O-glucoside (16) | 3.99 | 2.24 | Francisco et al, 2009^5^ |
| 6 | quercetin-3-O-(sinapoyl)-sophoroside-7-O-glucoside (17) | 2.63 | 2.95 | Francisco et al, 2009^5^ |
| 7 | kaempferol-3,7-di-O-glucoside+ kaempferol-3-O-(sinapoyl)sophoroside-7- O-glucoside (5+20) | 5.01 | 7.36 | Francisco et al, 2009^5^ |
| 8 | isorhamnetin-3,7-di-O-glucoside+kaempferol-3-O-(feruloyl)sophoroside-7-O-glucoside (6+21) | 11.09 | 10.15 | Francisco et al, 2009^5^ |
| 9 | quercetin-3-O-(feruloyl)sophoroside (26) | 0.3 | 0.22 | Francisco et al, 2009^5^ |
| 10 | 3-caffeoyl quinic acid (3-CQA) | 0.75 | 0.75 | Francisco et al, 2009^5^ |
| 11 | 3-p-coumaroylquinin acid (3p-CoQA) | 3.41 | 2.55 | Francisco et al, 2009^5^ |
| 12 | sinapic acid (SA) | 12.46 | 2.14 | Francisco et al, 2009^5^ |
| 13 | 1,2-disinapoylgentiobioside (A1) | 1.43 | 1.57 | Francisco et al, 2009^5^ |
| 14 | 1-sinapoyl-2-feruloylgentiobioside (A2) | 3.19 | 2.47 | Francisco et al, 2009^5^ |
| 15 | 1, 2, 2` -trisinapoylgentiobioside (A3) | 0.39 | 0.44 | Francisco et al, 2009^5^ |
| **Organic acids** | | | | |
| 16 | oxalic acid | 138.40 | 46.65 | Arias-Carmona et al ^8^ |
| 17 | malic acid | 89.34 | 37.12 | Arias-Carmona et al ^8^ |
| 18 | **citric acid** | **56.75** | **101.42** | Arias-Carmona et al ^8^ |
| 19 | ascorbic acid | 37.13 | 63.47 | Arias-Carmona et al ^8^ |
|  |  | 62 | 46 | Francisco et al, 2010^6^ |
|  | | **Turnip leaves and stems, mg/100g dw** | |  |
| 20 | Aconitic acid | 16.08-1247.2 | | Fernandes at al, 2007^9^ |
| 21 | Citric and ketoglutaric acids | 524.12-1856.5 | | Fernandes at al, 2007^9^ |
| 22 | Malic acid | 1880.0-3929.6 | | Fernandes at al, 2007^9^ |
| 23 | Shikimic acid | 1.9-30.33 | | Fernandes at al, 2007^9^ |
| 24 | Fumaric acid | 39.13-168.35 | | Fernandes at al, 2007^9^ |
|  |  |  | |  |

|  |  |  |
| --- | --- | --- |

**References**

1. US Department of Agriculture ARS. Nutrient Data Laboratory. USDA National Nutrient Database for Standard Reference, Release 28 (Slightly revised). Version Current: May 2016. <http://www.ars.usda.gov/nea/bhnrc/mafcl>.

2. Francisco M, Cartea ME, Soengas P, et al. Effect of genotype and environmental conditions on health-promoting compounds in brassica rapa. *Journal of Agricultural and Food Chemistry* 2011;59(6):2421-31. doi: 10.1021/jf103492r

3. Klopsch R, Witzel K, Borner A, et al. Metabolic profiling of glucosinolates and their hydrolysis products in a germplasm collection of Brassica rapa turnips. *Food Res Int* 2017;100(Pt 3):392-403. doi: 10.1016/j.foodres.2017.04.016 [published Online First: 2017/10/02]

4. Padilla G, Cartea ME, Velasco P, et al. Variation of glucosinolates in vegetable crops of Brassica rapa. *Phytochemistry* 2007;68(4):536-45. doi: 10.1016/j.phytochem.2006.11.017 [published Online First: 2006/12/26]

5. Francisco M, Moreno DA, Cartea ME, et al. Simultaneous identification of glucosinolates and phenolic compounds in a representative collection of vegetable Brassica rapa. *Journal of Chromatography A* 2009;1216(38):6611-19. doi: 10.1016/j.chroma.2009.07.055

6. Francisco Marta VP, A.Moreno Diego, García-Viguera Cristina, Carteaa María Elena. Cooking methods of Brassica rapa affect the preservation of glucosinolates, phenolics and vitamin C. *Food Research International* 2010;43(5):1455-63

doi: <https://doi.org/10.1016/j.foodres.2010.04.024>

7. Cartea ME, de Haro A, Obregón S, et al. Glucosinolate Variation in Leaves of Brassica rapa Crops. *Plant Foods for Human Nutrition* 2012;67(3):283-88. doi: 10.1007/s11130-012-0300-6

8. Arias-Carmona Dolores M R-RÁM, Vázquez-Odériz Lourdes M. Determination of Organic Acids in Brassica Rapa L. Leaves (Turnip Greens and Turnip Tops) Regulated by the Protected Geographical Indication “Grelos De Galicia”. *Journal of Food and Nutrition Research* 2014;2(11):786-91

doi: 10.12691/jfnr-2-11-5

9. Fernandes F, Valentão P, Sousa C, et al. Chemical and antioxidative assessment of dietary turnip (Brassica rapa var. rapa L.). *Food Chemistry* 2007;105(3):1003-10. doi: 10.1016/j.foodchem.2007.04.063
